# A New Laboratory Radio Frequency Identification (RFID) System for Behavioural Tracking of Marine Organisms

**DOI:** 10.3390/s111009532

**Published:** 2011-10-11

**Authors:** Jacopo Aguzzi, Valerio Sbragaglia, David Sarriá, José Antonio García, Corrado Costa, Joaquín del Río, Antoni Mànuel, Paolo Menesatti, Francesc Sardà

**Affiliations:** 1 Instituto de Ciencias del Mar (ICM) del Consejo Superior de Investigaciones Científicas (CSIC), Paseo Maritimo de la Barceloneta, 37–49, 08003 Barcelona, Spain; E-Mails: sbragaglia@icm.csic.es (V.S.); jagarcia@icm.csic.es (J.A.G.); siscu@icm.csic.es (F.S.); 2 Centro de Desarollo Tecnológico de Sistemas de Adquisición remota y Tratamiento de la Información (SARTI) de la Universitat Politècnica de Catalunya (UPC), Rambla de la Exposición 24, 08800 Vilanova i la Geltrú-Barcelona, Spain; E-Mails: david.sarria@upc.edu (D.S.); joaquin.del.rio@upc.edu (J.R.); antoni.manuel@upc.edu (A.M.); 3 AgritechLab-Agricultural Engineering Research Unit (ING) of the Agriculture Research Council (CRA), Via della Pascolare, 16, 00015 Monterotondo Scalo-Rome, Italy; E-Mails: corrado.costa@entecra.it (C.C.); paolo.menesatti@entecra.it (P.M.)

**Keywords:** RFID, automated video imaging, *Nephrops norvegicus*, controller, USB communication, marine species, laboratory, burrow emergence, activity rhythms

## Abstract

Radio frequency identification (RFID) devices are currently used to quantify several traits of animal behaviour with potential applications for the study of marine organisms. To date, behavioural studies with marine organisms are rare because of the technical difficulty of propagating radio waves within the saltwater medium. We present a novel RFID tracking system to study the burrowing behaviour of a valuable fishery resource, the Norway lobster (*Nephrops norvegicus* L.). The system consists of a network of six controllers, each handling a group of seven antennas. That network was placed below a microcosm tank that recreated important features typical of *Nephrops’* grounds, such as the presence of multiple burrows. The animals carried a passive transponder attached to their telson, operating at 13.56 MHz. The tracking system was implemented to concurrently report the behaviour of up to three individuals, in terms of their travelled distances in a specified unit of time and their preferential positioning within the antenna network. To do so, the controllers worked in parallel to send the antenna data to a computer via a USB connection. The tracking accuracy of the system was evaluated by concurrently recording the animals’ behaviour with automated video imaging. During the two experiments, each lasting approximately one week, two different groups of three animals each showed a variable burrow occupancy and a nocturnal displacement under a standard photoperiod regime (12 h light:12 h dark), measured using the RFID method. Similar results were obtained with the video imaging. Our implemented RFID system was therefore capable of efficiently tracking the tested organisms and has a good potential for use on a wide variety of other marine organisms of commercial, aquaculture, and ecological interest.

## 1. Introduction

Radio frequency identification (RFID) technology consists of the remote identification of objects that bear specific tags, which transmit individualised information to antenna receivers [1]. Currently, this technology is widely used in industrial chains for the processing and distribution of raw materials or derived products [2]. RFID technology has been recently applied to the behavioural tracking of animals over different temporal and spatial scales [3,4]. In fact, in the past 20 years, most of the RFID technological development consisted of the implementation of devices for the remote locating and counting, not only of livestock with a relatively limited range of displacement, but also of bees and birds with free movements [5,6]. Currently, there are no similar applications for small animals in confined laboratory environments, although RFID research is progressively moving to applications in indoor closed spaces [7].

In laboratory neuroscience, there is a constant need for tools to remotely and non-invasively monitor the behaviour of animals. Monitoring is especially important for chronobiology, the science of biological rhythms [8] because the continuous measurement of the behaviour of laboratory animals is the primary step in the characterisation of the mode of functioning of their biological clocks [9]. In rats, mice or hamsters, laboratory experiments on behavioural rhythms can be carried out using battery-free implantable telemetric transponders, such as the Mini Mitter, which allow a signal to be received in a 30 cm horizontal and 20 cm vertical field (http://www.minimitter.com/animal_products.cfm). No similar commercial and proven technology is available for tracking marine organisms. In fact, telemetry is inefficient in saltwater because of the attenuation that radio waves undergo within the medium, and the laboratory tools available for the study of behavioural characteristics of marine organisms are instead chiefly based on automated video imaging, weight balance platforms, and infrared barrier tracking technologies [10].

To the best of our knowledge, RFID monitoring of the behavioural activities of marine animals in laboratory facilities has not yet been reported. RFID tracking technology may be important for the management of commercially important marine resources, which requires a preliminary laboratory study of their day-night activity rhythms. Currently, the biomass assessment of several commercial marine demersal (*i.e.*, bottom-living) resources with a burrowing or tunnel-making life habit is limited by their daily behaviour. This limitation is true in the case of the Norway lobster, *Nephrops norvegicus* (L.), one of the most important commercial fishery items in Europe [11]. *Nephrops* inhabits complex burrow systems in the muddy continental shelves and slopes. These structures are used for shelter during times of inactivity and are the object of a strong territorial behaviour [12]. In the field, this species shows massive day-night population burrow-emergence rhythms that may provoke biases in the demographic assessment using trawl hauling [13,14]. In fact, animals can be captured by trawl nets only when they are outside their burrows [15].

Reliable estimations of the *Nephrops* population biomass are difficult without a comparison with behavioural laboratory tests [16]. A link between the knowledge collected in the laboratory on the inter-individual variability in burrow emergence rhythms and the uncertainties concerning the estimation of *Nephrops* biomass has not yet been shown. This link is currently under investigation. In this context, a potential research scenario may focus on sociality, including aggressive interactions as a putative modulator of the predisposition of animals to burrow emergence. Unfortunately, laboratory tests to date have been chiefly conducted on isolated individuals, and the results have been used to interpret the catchability dynamics of the population in the field with little success [10,17]. Therefore, laboratory tests with groups of animals seem to be necessary to produce results that are more relevant to the field context. Accordingly, RFID technology could be a valuable tool for behavioural tracking in laboratory tests with a group of *Nephrops* held within a microcosm tank containing multiple burrows. The resulting datasets may consist of a time series of consecutively gathered position data for each individual, presented as coordinates of the individual’s location [18]. In this work, we present a novel RFID tracking system for the characterisation of burrow emergence rhythms in a group of *Nephrops*. Our aim is to present a novel tool for behavioural monitoring as a complement to currently available technologies, such as IR actography or automated video imaging [19].

## 2. Animal Collection and Acclimation

Animals were collected on the continental margin shelf at approximately 80 m of depth off the Ebro delta (Spain) in the northwestern Mediterranean (between 40°39′N, 1°13′E and 40°38′N, 1°11′E) in February 2009. It has been previously shown that fishing and pressure changes do not influence the behaviour and underlying physiology of *Nephrops* and that their health status is usually good at the time of deck sorting from the net [16]. To avoid retinal damage upon exposure to the sunlight [20], the trawl sampling and all of the hauling operations were performed at night under dim red light (*i.e.*, intensity equal to 1 lx). After the net sorting, the animals were immediately transferred to dark, refrigerated containers with constant aeration of the water. An acclimation was carried out in a lightproof isolated chamber for one month prior to the experiments. The acclimation conditions simulated the species’ habitat requirements at the depth of sampling: (i) a constant temperature of 13 ± 0.1 °C [21]; (ii) monochromatic blue (480 nm) light-darkness regimes of 10 lx; (iii) standard 12-h photophase duration, starting at 7:00 h and ending at 19:00 h; and (iv) progressive attainment of a maximum light intensity within 30 min to acclimatise the lobsters’ eyes while avoiding optical damage [20]. Recently, Aguzzi *et al.* [22] and Chiesa *et al.* [23] showed that monochromatic 480-nm blue light regulates the daily activity rhythms of animals from shelves and slopes, and this wavelength of radiation is invariantly present throughout the twilight zone [24].

## 3. System Architecture

### 3.1. The Microcosm Tank

**Figure 1.**
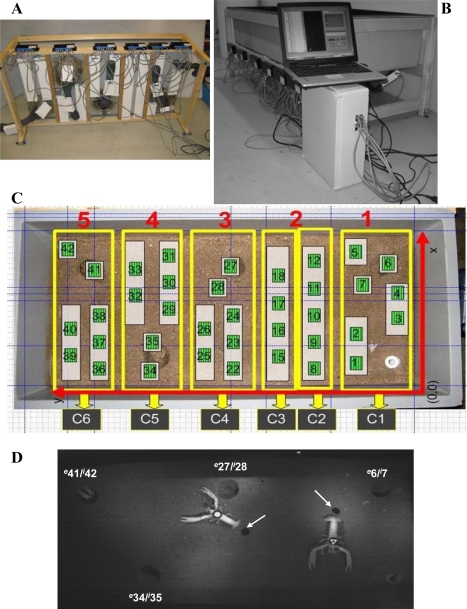
Details of the RFID system used to monitor the behaviour of a group of *Nephrops* held in a laboratory microcosm tank. **(A)** Details of the antenna network (the large white plates) mounted below the microcosm tank, including those placed on the tunnels (the small white plates on the grey tubes). **(B)** A side view of the tank showing the laptop used to acquire the RFID data from the controllers. **(C)** An upper view of the tank superimposed with the scheme depicting the antenna positioning (green squares numbered from 1 to 42); the antenna relationship with the controllers (from C1 to 6), covering five different areas (from 1 to 5) of the tank; and the *x*,*y* reference coordinates used to compute the animals’ displacement in centimetres. **(D)** A video camera field of view encompassing the whole tank surface, where the following objects are visible: the four burrows indicated by their antennas (numbers) placed inside (*^i^* = inside) or at the entrance (*^e^* = entrance); two fully emerged and one partially sheltered animal dragging their black circular RFID transponders, indicated by the white arrows; and finally, the geometric tags used for the automated video imaging.

A polycarbonate microcosm tank of 150 cm × 70 cm × 30 cm was constructed to simulate selected environmental features of a typical *Nephrops*’ habitat, see Figure 1(A,C). The burrow entrance and tunnel diameters were 10 cm and 7 cm, respectively. The tunnel length was 25 cm, with an approximately 20° angular inclination of the burrow entrance. A substratum simulating the muddy-sandy sediment was also added. This substratum was made of synthetic acrylic and was glued to the tank base.

### 3.2. The RFID System

RFID tracking systems usually comprise the following components: (i) a transponder, which stores and transmits, via radio waves, the identifying information of the carrier; (ii) a reader, *i.e.*, the antenna, which emits radio signals and in return receives answers from the transponder; and (iii) a central unit that controls and processes the spatiotemporal information of the transponder positioning [25,26]. In agreement with this organisational scheme, our RFID tracking system (Figure 1) was composed of a set of antennas that were equidistantly deployed immediately beneath the bottom of the microcosm tank to maximise the coverage of its surface, see Figure 1(C). The animals dragged a passive transponder that transmitted its unique identifier (UID) to the closest antenna when entering into the antenna’s powering field. The transponders were glued to a nylon cable (0.5 cm length) attached to the terminal segment of a lobster’s telson, see Figure 1(D). The antenna deployment in a grid allowed for the constant identification and displacement tracking of the animals at a centimetre scale.

The propagation of radio frequency signals through water is very different from their propagation through air because of differences in the permittivity and electrical conductivity of the medium. Wave attenuation in water is high compared to the attenuation in air and increases rapidly with increasing frequency. Because of the strong attenuation of radio waves in seawater and the reduced size of the animals, we tested transponders of different sizes, and therefore traceability, prior to starting the experiments. The operative factors we considered when choosing the type of antenna were anti-collision (*i.e.*, the detection of multiple transponders at the same time by an antenna) and the transponder/antenna size relationship (*i.e.*, the decreases in transponder size with an increase in the frequency, improving the locating accuracy of each antenna). Although we obtained the best detection results in terms of coverage using a frequency of 125 kHz, we selected an operating frequency of 13.56 MHz to improve the resolution and accuracy of locating the animals. A preliminary comparative study (Table 1) was conducted to ascertain the level of coverage of different antennas according to the different sizes and emitting capabilities of transponders at 13.56 MHz. That study was done within our distance specifications of the thickness of the tank. A round transponder with a 3.0 cm diameter and a weight of 0.646 g was chosen to cover a minimum tracking diameter of 6.0 cm.

**Figure 2.**
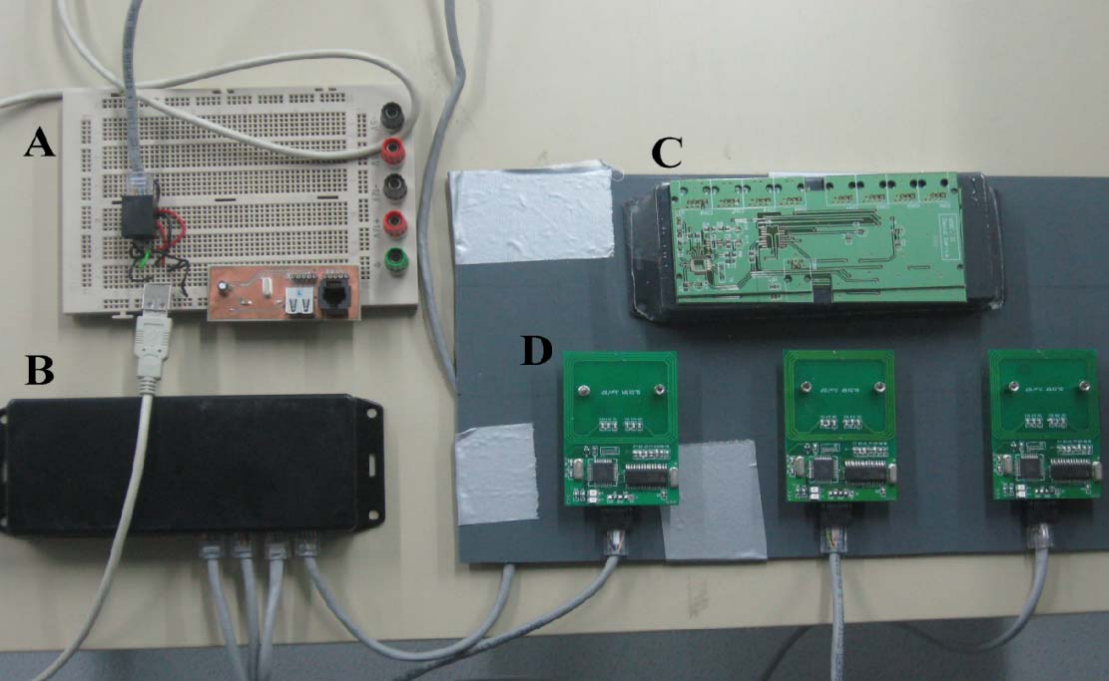
The components of the RFID system used to individually track the behaviour of *Nephrops* within a laboratory microcosm tank. The different elements are **A**, the USB to RFID controller interface; **B**, the RFID controller; **C**, the RFID controller electronic board in detail; and **D**, the antennas.

We used a low cost modular controller (Figure 2) to cyclically communicate with a row of seven antennas in a consecutive fashion. The controller managed the antennas through a unique universal asynchronous receiver transmitter (UART) port for the serial communication interface. Quick multiplexers were used to connect each antenna to the UART module.

**Figure 3.**
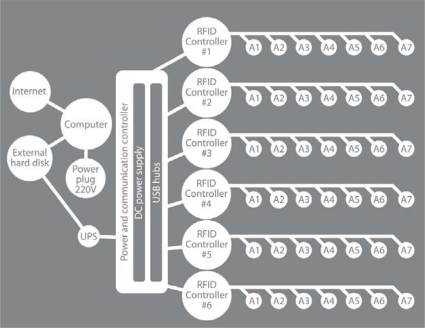
Organisational scheme of the relationships among the different elements composing the RFID system used to track behavioural rhythms in a group of *Nephrops* within a laboratory microcosm tank. The system is composed of the six controllers, each potentially connectable to seven antennas (A) and a signal storing tool (*i.e.*, the laptop) connected via USB hubs. Although 42 antennas could have been installed, our tank specifications required us to modify this design and to reduce the number of antennas that were connected to controllers 2 and 3, see Figure 1(C).

We installed six controlling units and 37 antennas. As shown in Figure 1(C), the control units C1, C4, C5, and C6 managed seven antennas, control unit C2 managed five antennas, and control unit C3 only managed four antennas for a total of 37 readable antennas (Figure 3). Every controller worked in parallel with each of the others, recording the positions of all of the animals every 5 seconds. The controllers worked with a time routine that consisted of the connection to the next antenna when the current one did not answer. All of the controllers swept all of their antennas in sequence to determine their status. An animal’s position could be directly extracted from the known location of the antenna.

The UART module connected to the onboard USB converter when the controllers ended the sweeping of all of their antennas. All of the stored information from tracking the animals was then sent to the computer through the USB interface. A LabVIEW application managed the data acquisition and storage of the data from the antennas. The application stored the position of every animal in a file when an antenna detected the passage of a transponder. A remote server application was used to control and monitor the progress of the experiments. The experimental data could be downloaded from a file transfer protocol (ftp) server without interrupting the data acquisition.

Because each antenna needed 150 mA at 5 V to detect a transponder and because the experiments were performed with three animals, the energy requirements exceeded the capacity of the USB when all of the antennas of the same controller detected transponders [27]. Therefore, the antennas were not powered by the USB power lines. We developed a second system to power all of the antennas using USB hubs and a DC power supply. For the same reason, the cable that connected the controllers with the high-level control system was a customised cable that was capable of bearing the power load of all of the antennas.

### 3.3. The Video Image-Tracking System

To test the tracking efficiency of the implemented RFID system, an automated video analysis was carried out on images acquired at a 5-s frequency, using the time-lapse mode of the camera, during the RFID tracking experiments. A digital camera (UI-1545LE-M, IDS), with an infrared USB 2.0 monochrome high-quality CMOS sensor, took images at a 1,280 pixels × 1,024 pixels resolution (SXGA/1.3 MP), controlled using the uEye Trigger (Imaging Development Systems-IDS; GmbH) software application. The video camera was equipped with a wide-angular objective of 6.0 mm, an F1.4 screw C 1/2 (IDS) lens, and a polarised filter. The camera was placed on a tripod at 1.5 m directly above the microcosm tank in a central position over its surface, see Figure 1(D). Two infrared lights of 150 W each (Infraphil PAR38E 150W E27 230V 1CT; Philips) were placed on both sides of the video camera at a distance of 20 cm to allow video filming during the periods of darkness.

The automated video imaging was carried out according to Menesatti *et al.* [28]. Briefly, three different geometric tags [a triangle, an outlined triangle, and a circle; see Figure 1(D)] were used to identify the animals. These tags were fixed on the superior part of the animals’ cephalothorax with fast-acting glue, which was removable at the end of the experiment with no damage. A customised script was developed to elaborate the images with the Image Processing Toolbox of Matlab 7.1 (The Math Works; Natick, MA, USA.). The automation operated according to the following steps: (i) tag detection by thresholding and image subtraction, which occurred against a background reference image of the tank without animals; (ii) binary segmentation of the subtracted product by grey-level thresholding, which consisted of the elimination of the pixels with grey levels >10 for night and >20 the day; and (iii) size filtering by removing extraneous objects in the area outside of the pixel range 100–400. The morphometric recognition of the extracted tags was performed with a shape-matching procedure, which was based on the *a priori* construction of a geometrical tag form to be superimposed for matching onto the tracked tag using a progressive angular rotation in 2° steps. The tag coordinates between consecutive frames were then computed as Euclidean distances and transformed into centimetres within the *x*,*y* reference system given by the tank size, see Figure 1(C).

### 3.4. Behavioural Experiments and Data Analysis

Two behavioural tracking experiments were carried out using three different adult inter-moult males of a similar size, with a mean carapace length ± sd of 46.17 ± 6.17 mm. Inter-moult individuals were chosen because moulting would eventually lead to the loss of the RFID tag. The first experiment lasted from 24 to 31 March 2009 (*i.e.*, 8.3 days). The second experiment lasted from 17 to 23 April 2009 (*i.e.*, 7.2 days). The experiment duration was set as one week because a week is usually an acceptable temporal duration of behavioural datasets to carry out a reliable chronobiological analysis of day-night rhythms [9].

The datasets of the animal positions from the RFID system and automated video imaging were analysed using current chronobiological statistical methods, conducted in parallel to compare their efficiency. A similar comparison was carried out to test the efficiency of our customised RFID system for identifying burrow emergence rhythms. The obtained RFID and video-imaging time series were named after the animals’ cephalothorax geometric tag, see Figure 1(D).

The time series analysis was carried out using the software package *El Temps* (elaborated by Díez-Noguera, University of Barcelona, Spain). First, the RFID and automated video-imaging displacement data (in centimetres) were binned in 10 min intervals. The binning was required prior to the data analysis to filter out the high-frequency noise typically present in time series of behavioural data [29]. Second, the data were represented over the experimental time, and the total activity was calculated for each animal as the sum of all of its displacements during the duration of the experiment. Third, the time series were screened using the periodogram developed by Sokolove and Bushell [30], between 20 and 27 h, to seek for the presence of 24-h based burrow emergence rhythms. In the output plots, peaks crossing the significance level (p > 0.05) indicated a significant period value.

Finally, a waveform analysis was performed to assess the phase of the rhythms, as the timing of the peaks in relation to the light intensity cycle. That analysis consisted of producing averaged activity profiles on a standard 24-h interval from each dataset. Waveforms were then computed as follows: the time series were subdivided into subsets of 144 values corresponding to 24 h; then, the subseries were averaged over each 10 min interval. The resulting mean curve, the waveform, was presented over 24 h along with the standard error of the mean (SEM) values. The significant activity peaks were determined by computing the midline estimating statistic of the rhythm (MESOR; [31]). The MESOR was calculated by re-averaging all of the waveform values to produce a horizontal line that was superimposed onto the waveform plot as a threshold. The phase duration and temporal limits were represented by waveform values above the MESOR. To identify tracking sensibility differences between the RFID and automated video-imaging, for each animal, both of the waveforms were plotted together to identify similarities in the profiles, in terms of the peak timing and phase onset and offset (*i.e.*, values above and below the MESOR).

Finally, we used the RIFD tracking data to identify the occurrence of burrow occupancy by the animals and any preferential stationing in different areas of the tank during the experiments. That operation was carried out to show the occurrence of territorial behaviour, another potential factor of biological interest measurable using our tracking method. To do so, the total number of detections per antenna was estimated with LabView separately for each animal. The resulting individual files were edited using LibreOffice Calc to generate plots of the total percentages of area occupation per each antenna. The data were presented considering the tank surface and referencing coordinates, see Figure 1(C).

## 4. Results

### 4.1. Burrow Emergence Rhythms

**Figure 4.**
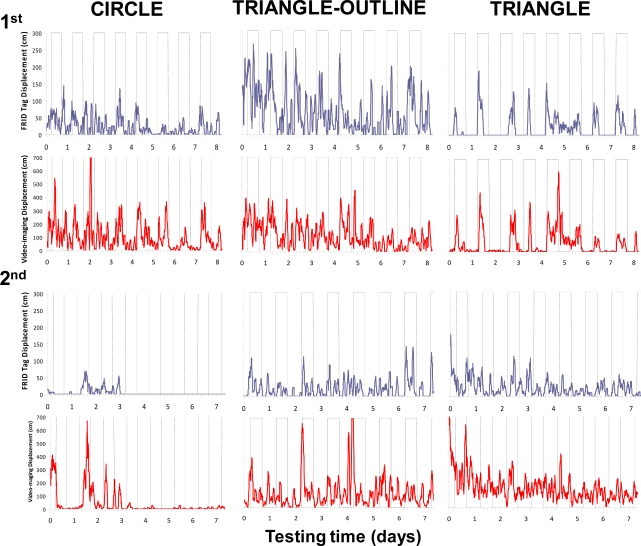
Time series of recurred distances by 6 individuals of *Nephrops* tracked by RFID (blue) and automated video imaging (red) in a day-night photoperiod regime (vertical grey rectangles indicate the night duration), during the first (1st) and the second (2nd) experiment.

The RFID tracking system successfully produced a time series of displacement data that could be used to determine locomotor activity rhythms and burrow emergence patterns (Figure 4). The RFID system assessed the occurrence of increases in the nocturnal activity of variable amplitude across days. The tracked displacements also varied among the animals (Table 2). In fact, we observed the presence of a strong inter-individual variability in the total displacement activity.

The RFID system had a different sensibility than the video tracking (see Table 2). The total movement computed for each specimen during the whole duration of the test was higher when measured using the automated video imaging. Despite this, the video-imaging time series showed a pattern similar to that obtained using the RFID tracking.

**Figure 5.**
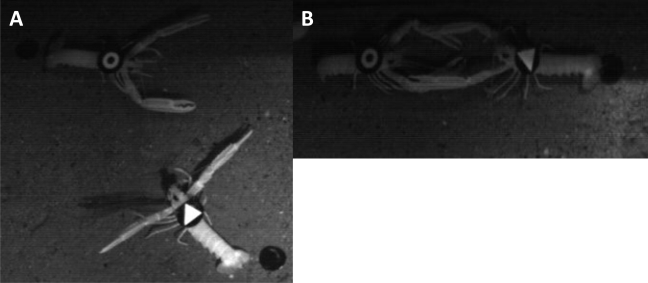
A photographic sequence of aggressive interactions between two individuals of *Nephrops* is presented as an example of the mechanism that generates burrow emergence rhythms of different strengths. The initial aggressive postures **(A)** during the approach of the animals are indicated by the forward maximum opening of the claws. This aggressive display is followed by a short contact confrontation **(B)**. The geometric tags for the automated video imaging and the dragged black RFID transponders are also visible.

The occurrence of a variable strength in the reported rhythmic locomotor patterns by both the RFID and automated video-imaging tracking systems could be ascribed to aggressive territorial behaviour, although this suggestion merely represents an operative hypothesis at this stage of research because the dataset is limited (Figure 5). We observed territorial interactions, which usually consisted of aggressive postures with the animals facing each other and projecting their claws forward at a 45° angle. That behavioural sequence was repeated several times within an encounter. Encounters of that kind were repeated over the days, ultimately resulting in the decrease in the rate and range of displacement of one of the animals. For example, the RFID time series for the “triangle” animal showed lower activity levels during the first experiment than those reported for both the “triangle-outline” and the “circle” individuals (see Table 2 and Figure 4). Both of these two latter animals showed a weaker burrowing behaviour, apparently being more often engaged in wandering out of their burrows.

A periodogram analysis (Table 3) detected the occurrence of similar significant periodicities in the locomotor activity rhythms in both of the RFID and automated video-imaging time series. This similarity was considered as a marker of the precision of the implemented RFID tracking methodology as compared to the automated video imaging. Significant period values indicated the occurrence of diel rhythms in all of the animals in the first experiment, while arrhythmia was reported in some of the animals in the second experiment. That arrhythmia could be due to the low activity of the animals, which can be observed in the time series of Figure 4.

**Figure 6.**
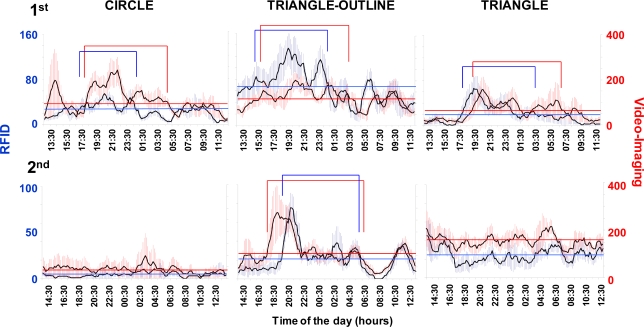
Comparisons of waveform analysis outputs between locomotion activity time series (cm ± SEM) that were measured over the first (1st) and second (2nd) day-night experiment using RFID (blue) and automated video-imaging (red) tracking methods for three *Nephrops* held together in a laboratory microcosm tank. The MESOR is the horizontal line. The arrows indicate the onset and offset of peaks (*i.e.*, values above the MESOR) and are used for a comparison between the two tracking methods.

The peak timings, or timings of the values above the MESOR, were compared among the waveform analysis (Figure 6) output plots. The average activity profiles from the RFID and automated video-imaging time series showed consistent similarities. These similarities were visible by comparing the temporal onsets and offsets of the peaks. Generally, the lobsters’ waveform profiles showed a broad nocturnal activity, with the occurrence of the major peak at approximately sunset. The RFID and video-imaging profiles were usually also similar in cases where no clear phase definition could be carried out, such as in the case of the triangle animal in the second experiment. Again, this fact was considered as an indication of the tracking accuracy of the implemented RFID system.

**Figure 7.**
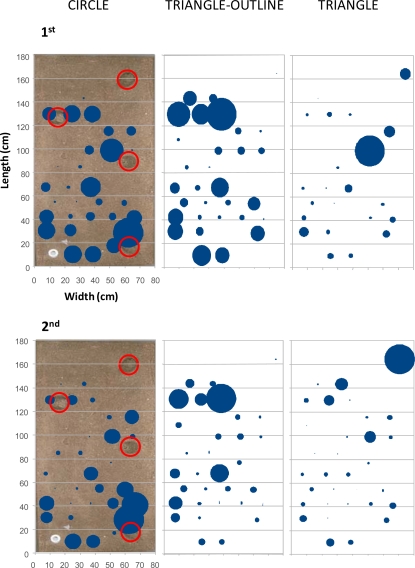
Inter-individual differences in the occupied areas of a group of three *Nephrops*, detected using RFID tracking during the first (1st) and second (2nd) day-night experiment. The blue circles are proportional to the percentage of occupation, computed from the cumulative detections in each antenna field over the duration of the experiment. The red circles indicate the entrances of the four tunnels, with their location superimposed onto a picture of the tank area for which the length and width coordinates have been also reported as a system of *x*,*y* coordinates, see Figure 1(C).

In Figure 7, the outputs of the percentage of occupation analysis are represented. The percentage of occupation was calculated from the presence of each animal within each antenna field during the whole duration of the experiments. That analysis was performed to show the potential of our RFID tracking system for assessing the overall inter-individual differences in area occupation related to the presence of burrows. The activity was more spatially diffused in the first experiment, with only one animal, the triangle, located in a preferred area, the one close to the burrow at an intermediate distance on the right. A clearer differential distribution of activity was instead visible during the second experiment where, although the displacements were reduced (see Figure 4), the circle animal was often located in the bottom right corner of the tank, and the triangle animal mostly occupied the right superior zone.

## 5. Discussion

In this study, we have developed and tested a novel RFID tracking technology to measure the burrow emergence rhythm of a commercially important marine crustacean species. The tracking method was designed to study the behaviour of a bottom-dwelling species, and the method could be used to track the behaviour of any other motile organism, with the organism’s size as the only limitation because the organism must be able to bear the load of an RFID tag. The controllers were implemented with a USB interface to send the tracking data to the computer. Our modular RFID controller/antenna design presents a distributed character, which allows the potential system expansion if needed by simply connecting new controllers with their antennas to the USB ports. Currently, the number of controllers can be expanded up to 127 devices per port, based on the USB specifications. To our knowledge, the design used in this study may offer new tracking solutions for marine behavioural research.

Studies monitoring the behavioural activity of animals are important for the neuroscience field because data analysis can reveal how animal nervous systems function, including details regarding the regulation of the biological clock [8]. Unfortunately, some species can be sensitive to the tracking method used, a fact that may condition the outputs of behavioural monitoring. In our experimental tests, the dragging of the RFID tag did not seem to alter the animals’ behaviour or their free motility. In fact, the obtained results are similar to those reported in equivalent behavioural tests with animals that were free of any tail tag [28]. Therefore, we assume that our system is a suitable non-intrusive tracking tool for marine species within the size range of adult *Nephrops*. In smaller animals, the presence of RDIF transponder of a size similar to the one we used could consistently disrupt the animal’s temporal and spatial behavioural patterns.

Previous research on *Nephrops* locomotor rhythms was also carried out using the non-invasive methods of infrared actography of single individuals [19] or automated video imaging of groups [28]. Both of those methods may present difficulties when applied to groups of animals that can be solved by RIFD applications. Infrared actography requires detection barriers made by emitting and receiving LEDs to report a locomotion event as a shadow from an animal’s passage. Unfortunately, this design cannot be used in behavioural tests with groups of animals when the goal is to separately follow the activity rhythm of each animal. Conversely, automated video imaging allows the efficient tracking of the behavioural rhythms of a group of animals in an individualised fashion. This method provided higher total activity values (see Table 2) as visible in time series and waveforms (see Figures 4 and 6) than the RFID approach. Automated video imaging is more sensitive to movement tracking than RFID for detecting the movement of animals into the same antenna quadrant prior to their displacement into another quadrant. However, such a method does not provide burrow occupancy data and requires the constant tuning of image treatment protocols according to the different life habits of the targeted species. The software implementation in those cases may be very difficult depending on the contingent illumination conditions, such as the water surface reflection, and in the presence of transient artefacts, such as mobile food or moult leftovers, bubbles, detached body parts such as claws, and turbidity.

Generally, for behavioural research with aquatic organisms, the advantage of using RFID over video imaging is the capability of RFID to detect animals independently of the visibility conditions. However, automated video imaging was more sensitive than RFID to movement tracking. Although both RFID and automated video imaging can efficiently track animals’ displacement, the latter provides more reliable estimations of recurred distances. In fact, RIFD detections occur only when an animal moves among the quadrants of different antennas, while video tracking can perceive additional *in situ* movements that may occur between adjacent antennas. This difference was evident by comparing the RFID and video-imaging waveform profiles for each animal (see Figure 6): the latter showed peak onsets prior to the former as a marker of activity onset with very short-range displacements. Despite these tracking differences, our RFID system facilitated the characterisation of rhythmic behavioural patterns that were similar in their periodicity and phase to those reported using video imaging.

The majority of marine coastal and deep-water species have unknown activity rhythms [32]. Our technological effort was carried out in the context of the present needs of marine chronobiology. Currently, data on the biological clocks of commercially important marine species are scarce because of the absence of reliable monitoring approaches in the laboratory. In the case of *Nephrops*, laboratory research on the activity rhythms of individuals within a group is of interest to evaluate the proportions of arrhythmic animals, which may never emerge and are therefore not counted using ordinary sampling systems, such as hauling. Our tested *Nephrops* showed nocturnal burrow-emergence patterns of variable strength. The nocturnal emergence behaviour fits with temporal trawling data from the Atlantic and Mediterranean shelves at a depth range of 20–200 m, where the inhabiting populations display massive nocturnal-crepuscular emergence rhythms [15,33]. These rhythms are sustained by synchronous increases in the locomotor activity and underlying physiological state of the individuals constituting the populations, which result in excursions and hence in haul net capture [34–37]. The variability in the detected emergence rhythms seems to be the combined product of concurrent inter-individual differences in behaviour [16]. The dataset is still too limited to ascribe the variability of the emergence rhythms to social aggressive interactions at this stage of the research. To date, the effect of territorialism on emergence suppression or enhancement has not yet been quantified and merits further research using larger numbers of animals. Further improvements in the experimental design would be to combine animals of different sex to more realistically estimate the animals’ behaviour on the seafloor and therefore to improve the present inventories of lobster stocks. These operative scenarios are now possible not only using automated video imaging but also using the implemented RFID tracking technology.

## Figures and Tables

**Table 1.** Technical specifications of the RFID transponders (tags) emitting at 13.56 MHz, tested to track the behaviour of *Nephrops* in a laboratory microcosm tank.

**Tag characteristics**			**Maximum tag-to-antenna distance reading through water (cm)**
**Diameter (cm)**	**Height (cm)**	**Weight (g)**	

3.45	0.68	3.578	8.0
3.00	0.08	0.646	6.0
2.50	0.10	0.762	5.5
2.00	0.11	0.516	4.5
1.70	0.34	0.604	3.0

**Table 2.** Total locomotor activity (cm) measured using RFID and automated video imaging of three *Nephrops* held together in a laboratory microcosm tank during two consecutive one-week experiments.

**Experiment**	**Animals**	**Total activity**	
		**RFID**	**Video**

**1st**	*Circle*	27,743	124,455
	*Triangle-outline*	60,177	111,454
	*Triangle*	21,222	63,399
**2nd**	*Circle*	4,545	41,561
	*Triangle-outline*	21,561	116,337
	*Triangle*	25,328	158,622

**Table 3.** Outputs of a periodogram analysis (*i.e.*, the periodicity P in min and the percentage of variance V%, as a measure of the strength of detected rhythms) of the time series of locomotion data for three *Nephrops* held together in a microcosm tank, obtained using RFID and automated video tracking during two consecutive laboratory experiments.

**Experiment**	**Animals**	**RFID**		**Video**	

		P	%V	P	%V

**1st**	*Circle*	1,430	23.02	1,440	36.6
	*Triangle-outline*	1,435	25.63	1,440	25. 64
	*Triangle*	1,440	27.64	1,440	21.75

**2nd**	*Circle*	−	−	−	−
	*Triangle-outline*	1,440	38.09	1,435	40.23
	*Triangle*	1,375	20.85	−	−
